# Candidate Serum Biomarkers of Neural Injury and Neuroinflammation in Childhood Epilepsy: Comparison Between Controlled and Drug-Resistant Phenotypes

**DOI:** 10.3390/children13091190

**Published:** 2026-09-03

**Authors:** Sevim Türay, Merve Alpay, Mehmet Ali Sungur, Elif Meliha Sözbir, Nefise Arıbaş Öz, Çağatay Zamur

**Affiliations:** 1 Department of Pediatric Neurology, Faculty of Medicine, Düzce University, Düzce 81620, Türkiye; 2 Department of Biochemistry, Faculty of Medicine, Düzce University, Düzce 81620, Türkiye; 3 Department of Biostatistics, Faculty of Medicine, Düzce University, Düzce 81620, Türkiye; 4 Department of Pediatrics, Faculty of Medicine, Düzce University, Düzce 81620, Türkiye

**Keywords:** glial fibrillary acidic protein, nitric oxide, ubiquitin, childhood epilepsy, drug-resistant epilepsy, biomarker

## Abstract

**Highlights:**

**What are the main findings?**

- Serum NO-related metabolite levels, GFAP, and ubiquitin levels did not differ significantly between controlled and drug-resistant epilepsy groups in children.
- Serum NO-related metabolite levels showed a significant positive association with age exclusively in the drug-resistant epilepsy group, surviving Bonferroni correction.

**What are the implications of the main findings?**

- No statistically significant between-group differences were detected in single time-point measurements of the three candidate biomarkers.
- The age-related increase in NO-related metabolites in drug-resistant epilepsy may reflect differences in NO metabolism associated with disease-related factors; however, this finding requires confirmation in longitudinal studies.

**Abstract:**

**Background:** This study aimed to compare serum levels of nitric oxide (NO) related metabolites, glial fibrillary acidic protein (GFAP), and ubiquitin (UBI) between children with controlled epilepsy (CE) and drug-resistant epilepsy (DRE) of unknown etiology, and to investigate the associations between these biomarkers and electroclinical features. **Methods:** Eighty-five children aged 2–18 years with epilepsy of unknown etiology who had been receiving antiseizure treatment for at least six months were enrolled; 58 were classified as CE and 27 as DRE. Serum GFAP and UBI concentrations were measured using enzyme-linked immunosorbent assay, and serum NO-related metabolites were assessed using a Griess-based colorimetric assay. Between-group comparisons were performed using the Mann–Whitney U test, and associations between biomarkers and clinical variables were evaluated using Spearman rank correlation analysis. Bonferroni correction was applied for multiple comparisons. **Results:** Historical seizure frequency, comorbidity rate, and seizure detection on initial video-EEG differed significantly between the CE and DRE groups. However, serum NO-related metabolite, GFAP, and UBI levels did not differ significantly between the CE and DRE groups. Correlation analyses revealed a significant positive correlation between serum NO-related metabolite levels and age exclusively in the DRE group (ρ = 0.594, *p* = 0.001), which remained significant after Bonferroni correction. No such association was observed in the CE group. **Conclusions:** No statistically significant differences in the measured peripheral serum biomarkers were detected between the CE and DRE groups in this sample. The age-related increase in NO-related metabolites observed in the DRE group should be interpreted cautiously and does not establish progressive NO upregulation or cumulative neuroinflammatory burden. Studies incorporating a healthy control group, standardized sampling intervals, and longitudinal designs are needed to clarify the clinical utility of these biomarkers in childhood epilepsy.

## 1. Introduction

Epilepsy is one of the most common neurological disorders of childhood, affecting approximately 1% of the general population. Beyond seizures themselves, the condition carries wide-ranging consequences for cognitive, psychological, and social functioning, and is frequently accompanied by a range of comorbidities. Childhood-onset epilepsy may have long-term effects on cognitive, behavioral, and neurological development, particularly when seizures are frequent or severe. Importantly, the neuronal injury set in motion by seizure activity does not necessarily resolve once seizures are brought under control; long-term consequences may persist well into adulthood. At the cellular level, repeated seizures drive dynamic structural and functional changes, including axonal and dendritic remodeling, gliosis, and apoptosis [1].

Childhood represents one of the most critical windows of brain development. Although the young brain possesses a remarkable degree of neuroplasticity, early-onset epilepsy with frequent and severe seizures is associated with central nervous system dysfunction that can persist into adult life [2]. The burden extends beyond neurology: childhood epilepsy increases the frequency of cognitive and behavioral difficulties, interferes with academic achievement, and imposes a substantial economic and social cost on affected families [3].

Neuronal injury may both drive epileptogenesis and arise as a consequence of recurrent seizures. This bidirectional relationship underscores the clinical importance of reliable biomarkers. Identifying and monitoring neuronal damage through accessible, minimally invasive markers could support risk stratification, deepen our understanding of epilepsy comorbidities, and open new avenues for therapeutic intervention [4].

In recent years, peripheral blood biomarkers have attracted growing interest as non-invasive tools for assessing central nervous system injury. Ubiquitin (UBI) and ubiquitin-binding proteins are among the principal constituents of the neurotoxic protein aggregates observed in many neurodegenerative conditions. Disruption of UBI-mediated protein degradation has been closely linked to mitochondrial dysfunction and neurodegeneration, and ubiquitin signaling is now being actively investigated as a therapeutic target in neurological disease [5].

Nitric oxide (NO) is a small, diffusible molecule that serves as a neuromodulator and intracellular messenger in the central nervous system. Under physiological conditions, it participates in synaptic plasticity, neurotransmission, cerebral blood flow regulation, and the control of neuronal excitability [6]. During seizures, however, excessive NO production can contribute to oxidative and nitrosative stress, mitochondrial dysfunction, blood–brain barrier disruption, and neuroinflammatory cascades [7]. Repeated epileptic activity may therefore activate NO-related pathways through excitotoxic neuronal stimulation and glial inflammatory responses. That said, both proconvulsant and anticonvulsant effects of NO have been reported depending on seizure type, experimental model, and timing of measurement, and its precise role in epilepsy remains complex and context-dependent [8,9].

Glial fibrillary acidic protein (GFAP) is the principal intermediate filament protein of astrocytes, the most abundant glial cells of the central nervous system. It plays a key role in maintaining white matter integrity, supporting myelination, and preserving blood–brain barrier continuity, as well as contributing to the structural stability and motility of astrocytes themselves. Highly brain-specific, GFAP is released from neural tissue into the peripheral circulation during reactive astrogliosis. This process is triggered by inflammation, hypoxia, or blood–brain barrier disruption [10,11].

The ability to detect seizure-induced neuronal injury, astroglial activation, and progressive neural network remodeling through blood-based biomarkers could make a meaningful contribution to the diagnosis, prognostic assessment, and monitoring of epilepsy. Yet data on peripheral serum biomarkers of neuronal damage in childhood epilepsy remain limited, and the relationship between circulating biomarker levels and disease severity is not fully understood. Although some studies have reported biomarker elevations in the postictal period, findings in pediatric populations have been heterogeneous and sometimes contradictory. Against this background, the present study aimed to compare serum levels of UBI, NO, and GFAP between children with controlled and drug-resistant epilepsy of unknown etiology, and to explore their potential clinical value as rapid, accessible, and minimally invasive candidate markers of neural injury and neuroinflammation in this population.

## 2. Materials and Methods

### 2.1. Study Population

Children aged 2–18 years who had been receiving antiseizure medication (ASM) for at least six months and whose epilepsy was classified as being of unknown etiology according to the ILAE 2017 classification were eligible for inclusion [12]. All participants were under regular follow-up at our outpatient clinic. Patients with structural abnormalities on cranial imaging, secondary brain injury, neurometabolic disease, or any chronic systemic condition (including diabetes, obesity, autoimmune disease, malignancy, chronic kidney or liver disease, or inherited metabolic disorders) were excluded, as were those with a follow-up duration of less than six months.

Patient recruitment was conducted consecutively between July 2022 and March 2024 among children followed in our pediatric neurology outpatient clinic who met the predefined eligibility criteria. Although the total number of patients screened during the recruitment period was not systematically recorded, 96 potentially eligible patients were assessed for inclusion. Eleven patients were excluded before the final analysis: three because of hemolyzed serum samples, four because of inadequate follow-up, two because subsequent clinical findings raised suspicion of a genetic syndrome, one because of diabetes mellitus, and one because of Behçet disease. The final analysis therefore included 85 patients. No biomarker measurements were missing among participants included in the final analysis.

All participants underwent cranial MRI, and patients with structural abnormalities were excluded. Additional genetic, metabolic, immune, and infectious investigations were performed when clinically indicated based on clinical history, neurological examination, developmental profile, seizure characteristics, and other relevant findings. These investigations were therefore not uniformly performed in all participants. Epilepsy was classified as being of unknown etiology when no specific cause was identified through clinical evaluation, neuroimaging, and, where indicated, additional etiological investigations.

The study comprised two groups: 58 children with controlled epilepsy (CE) and 27 children with drug-resistant epilepsy (DRE). The CE group consisted of patients who achieved seizure freedom with a single ASM and fulfilled the ILAE seizure-freedom duration criterion, defined as seizure freedom for at least 12 months or three times the longest preintervention interseizure interval, whichever was longer [13]. The CE group was intentionally restricted to patients receiving a single ASM to reduce heterogeneity in ASM exposure and minimize potential medication-related confounding of serum biomarker concentrations. DRE was defined according to the ILAE consensus definition as failure of adequate trials of two tolerated, appropriately chosen and used ASM schedules, whether as monotherapies or in combination, to achieve sustained seizure freedom [13]. Treatment adequacy was determined based on the appropriateness of the ASM for the seizure and epilepsy type, tolerability, use at an adequate therapeutic dose, and sufficient treatment duration to permit assessment of therapeutic response.

Demographic and clinical characteristics were compared between groups. Data on family history, clinical findings, age at seizure onset, epilepsy duration, seizure semiology, historical seizure frequency, and current and prior ASM use were obtained through structured interviews and medical record review.

### 2.2. Serum Biomarker Measurements

Peripheral venous blood samples were collected into serum biochemical tubes, allowed to clot at room temperature, and centrifuged at 3000 rpm for 15 min at 4 °C. The separated serum samples were aliquoted and stored at −80 °C until analysis. Although patient recruitment extended from July 2022 to March 2024, blood samples used for biomarker analyses were collected during the final 6 months of the study. Samples underwent no more than one freeze–thaw cycle before analysis. Hemolyzed and lipemic samples were excluded prior to biochemical analyses.

The original study protocol included a healthy-control group of 30 children. Control samples were collected during the final months of the recruitment period and, for logistical reasons, were stored in a separate deep-freezer located in the pediatric outpatient clinic area. When these samples were retrieved for biomarker analysis, a malfunction of this freezer was identified and the control samples were found to have thawed. Because their integrity could not be assured, they were excluded from the biochemical analyses. The CE and DRE serum samples were stored in a separate deep-freezer, remained frozen until analysis, and were not exposed to the storage failure affecting the control samples.

Serum GFAP and ubiquitin (UBI) concentrations were measured using commercially available sandwich enzyme-linked immunosorbent assay (ELISA) kits (Nepenthe Research Technologies, Gebze, Türkiye; GFAP, Cat. No. NE010209401; UBI, Cat. No. NE010161801), according to the manufacturer’s instructions. Samples were analyzed in duplicate, and optical density was measured at 450 nm. For the GFAP assay, the manufacturer-reported standard range was 0.13–16 ng/mL, with an analytical sensitivity of 0.026 ng/mL and intra-assay and inter-assay coefficients of variation (CVs) of <8% and <10%, respectively. For the UBI assay, the analytical measuring range was 37.5–2400 pg/mL, with an analytical sensitivity of 5.13 pg/mL and intra-assay and inter-assay CVs of <8% and <10%, respectively. Standard curves were generated for each assay, with a curve-fitting performance of R^2^ ≥ 0.995. GFAP and UBI concentrations were expressed as ng/mL and pg/mL, respectively.

NO-related metabolites were assessed using a commercially available Griess-based colorimetric assay (Elabscience, Houston, TX, USA; Cat. No. E-BC-K035-M), according to the manufacturer’s instructions. Samples were analyzed without dilution as single measurements, and optical density was measured at 550 nm. The analytical measuring range was 0.97–700 µmol/L, with intra-assay and inter-assay CVs of 2.4% and 3.7%, respectively. The standard curve showed an R^2^ of 0.9987. As the Griess-based assay does not directly measure free NO, NO production was estimated indirectly through its stable oxidized metabolites. Results were expressed as µmol/L.

Samples were labeled and arranged according to clinical group by the clinical team before biochemical analysis; therefore, laboratory personnel were not formally blinded to group allocation. Detailed individual plate-allocation records were not available retrospectively.

Biomarker concentrations were compared between the CE and DRE groups. Correlation analyses were subsequently performed to evaluate the relationships between biomarker levels and clinical variables, including age, epilepsy duration, seizure frequency, EEG abnormalities, and comorbidities.

### 2.3. EEG Analysis

For the present analysis, EEG data were obtained from the initial 1-h video-EEG recordings performed as part of the patients’ clinical evaluation and available in their medical records. These recordings preceded the assessment of seizure control and did not represent EEG monitoring performed at the time of study enrollment or blood sampling. Video-EEG recordings were obtained using a Natus Neurodiagnostics system (Natus Medical Incorporated, Middleton, WI, USA), with electrodes placed according to the international 10–20 system [14]. Each recording lasted approximately one hour and encompassed both wakefulness and sleep, captured simultaneously with synchronized video in a dedicated recording room with an external storage system. During the recording, patients’ behavioral state and responsiveness to external stimuli were assessed by a physician or nurse whenever a clinical event occurred.

Seizures were classified by onset as focal, generalized, or unknown, and by semiology as motor or non-motor. EEG findings were reviewed for the presence of electroclinical or purely electrical seizures. Spike and seizure detection software was used continuously throughout each recording session.

### 2.4. Statistical Analysis

The normality of continuous variables was assessed using the Shapiro–Wilk test. Continuous variables are presented as median and interquartile range (IQR), and between-group comparisons were performed using the Mann–Whitney U test. Categorical variables were compared using Fisher’s exact test or the Pearson chi-square test with Yates’ correction, as appropriate, and are presented as frequencies and percentages. Spearman’s rank correlation coefficient was used to examine associations between serum biomarker concentrations and clinical variables, including age, epilepsy duration, seizure frequency, EEG abnormality, and comorbidity. Two separate Bonferroni corrections were applied to account for multiple comparisons. For the comparison of three serum biomarkers between the controlled epilepsy and drug-resistant epilepsy groups, the corrected significance threshold was set at *p* < 0.017. For the correlation analyses involving three biomarkers and five clinical variables (15 simultaneous comparisons), the corrected threshold was *p* < 0.0033. A two-tailed *p*-value of less than 0.05 was considered statistically significant for all other analyses. To formally compare the Spearman correlation coefficients between the two groups, Fisher’s r-to-z transformation was applied. In the correlation analyses, age and epilepsy duration were treated as continuous variables. EEG abnormality and comorbidity were coded as binary variables (0 = absent, 1 = present). Seizure frequency was coded as an ordinal variable with three levels (1 = rare, 2 = persistent, 3 = daily), reflecting a clinically meaningful rank ordering of increasing seizure burden. Correlation analyses were performed separately within each group as two independent multiplicity families, with Bonferroni correction applied within each group (corrected threshold: *p* < 0.0033 per family). An alternative conservative approach treating all 30 correlations as a single multiplicity family would yield a corrected threshold of *p* < 0.0017; the statistically significant age–NO correlation in the DRE group (*p* = 0.001) would survive correction under either approach. Influential observations in the correlation analyses were identified using Cook’s distance, with a threshold of 4/*n*. All statistical analyses were performed using IBM SPSS Statistics version 26.0 (IBM Corp., Armonk, NY, USA).

## 3. Results

There were no significant differences between the groups regarding sex, age, birth weight, maturity status, NICU admission history, family history of febrile convulsions, family history of epilepsy, or consanguinity (*p* > 0.05 for all). However, comorbidities were significantly more frequent in the DRE group compared to the CE group (70.4% vs. 36.2%, *p* = 0.003). In addition, the distribution of comorbidity subtypes differed significantly between the groups (*p* = 0.004), mainly due to the higher rate of multiple comorbidities in the DRE group (Table 1).

Historical seizure-frequency distribution differed significantly between the groups (*p* < 0.001), with persistent and daily seizure patterns being more common in the DRE group. In addition, seizure classification and seizure semiology showed significant differences between the groups (*p* = 0.040 and *p* = 0.030, respectively). Seizure detection on the initial video-EEG also differed significantly between the groups (*p* < 0.001), with electroclinical and electrical seizures being more frequently identified in the DRE group (Table 2).

Current ASM exposure differed substantially between the groups, consistent with the predefined group characteristics. All 58 participants in the CE group were receiving monotherapy, whereas all 27 participants in the DRE group were receiving polytherapy with at least three ASMs. Among patients with DRE, 25 (92.6%) were receiving three ASMs and 2 (7.4%) were receiving four ASMs, corresponding to a mean current treatment burden of 3.07 ASMs per patient. The most frequently used ASMs were valproate (39.7%), levetiracetam (36.2%), and carbamazepine (17.2%) in the CE group, whereas valproate (77.8%), levetiracetam (63.0%), clobazam (44.4%), topiramate (37.0%), and lamotrigine (29.6%) were the most frequently used agents in the DRE group. Detailed current ASM exposure by group is presented in Supplementary Table S1.

**Figure 1:**
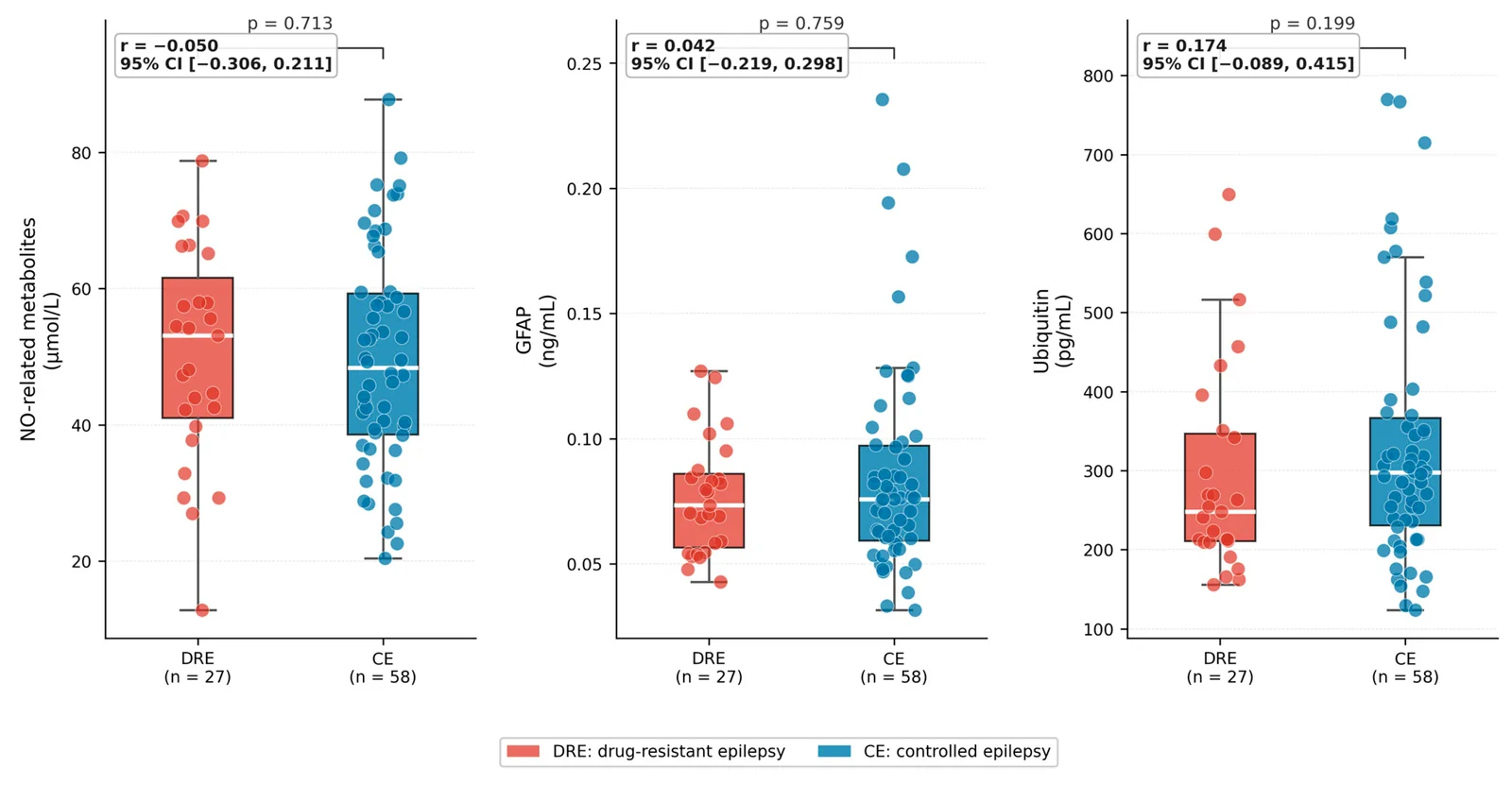
Box plots with individual data points showing serum concentrations of NO-related metabolites (µmol/L), GFAP (ng/mL), and ubiquitin (pg/mL) in the drug-resistant epilepsy (DRE, *n* = 27) and controlled epilepsy (CE, *n* = 58) groups. Horizontal lines within boxes represent medians; box limits represent the 25th and 75th percentiles; whiskers extend to 1.5 × interquartile range. Individual data points are shown as jittered dots. *p*-values were derived from the Mann–Whitney U test. Effect sizes are reported as rank-biserial correlation coefficients (r). NO, nitric oxide; GFAP, glial fibrillary acidic protein; DRE, drug-resistant epilepsy; CE, controlled epilepsy.

Serum biomarker concentrations did not differ significantly between the CE and DRE groups for any of the three markers measured (Table 3, Figure 1). All between-group effect sizes were small, with 95% confidence intervals crossing zero in all cases.

Values are expressed as median [IQR]. Between-group comparisons were performed using the Mann–Whitney U test with Bonferroni correction applied for three simultaneous comparisons (corrected threshold: *p* < 0.017). Effect size is reported as rank-biserial correlation coefficient (r) with 95% confidence intervals. All effect sizes were small (|r| < 0.30). Biomarker concentrations were derived from assay-specific standard curves. Abbreviations: NO, nitric oxide; GFAP, glial fibrillary acidic protein; UBI, ubiquitin; IQR, interquartile range; CI, confidence interval.

**Figure 2:**
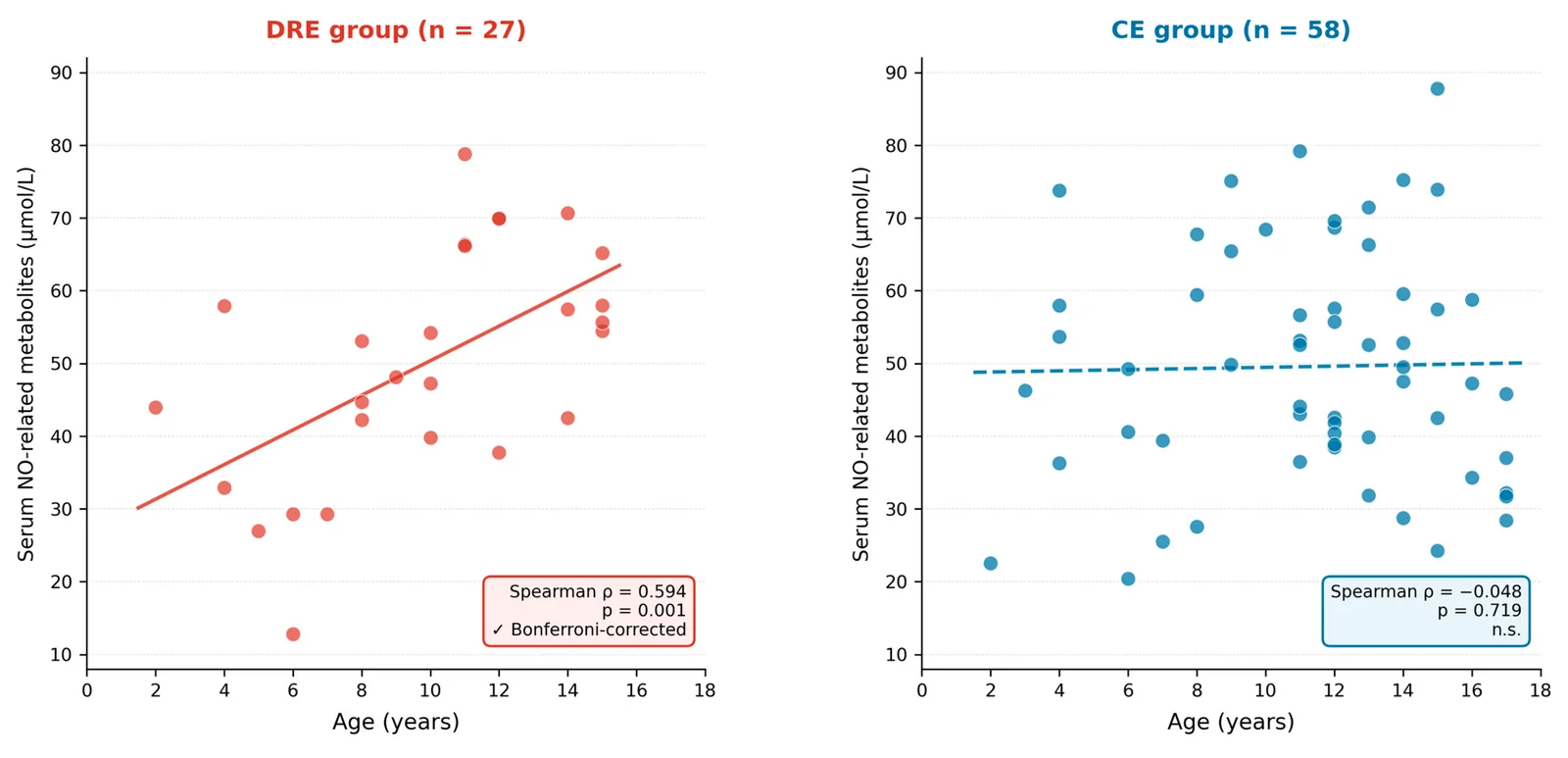
Correlation between serum NO-related metabolite levels and age in the drug-resistant epilepsy (DRE, left panel) and controlled epilepsy (CE, right panel) groups. Each point represents one patient. Trend lines represent linear fits for visual reference only. Statistical test: Spearman rank correlation. Bonferroni correction was applied for 15 simultaneous comparisons (corrected threshold: *p* < 0.0033). ✓ indicates Bonferroni-corrected significance. n.s., not significant; NO, nitric oxide; DRE, drug-resistant epilepsy; CE, controlled epilepsy.

Correlation analyses performed separately for each group revealed no significant associations between serum biomarker levels and clinical variables, including epilepsy duration, seizure frequency, EEG abnormalities, and comorbidities. However, in the DRE group, serum NO-related metabolite levels showed a significant positive correlation with age (ρ = 0.594, 95% CI: 0.276–0.795, *p* = 0.001), which remained significant after Bonferroni correction (Figure 2). No such association was observed in the CE group (ρ = −0.048, 95% CI: −0.303–0.213, *p* = 0.719). The correlation between age and serum NO-related metabolite levels was statistically significantly stronger in the DRE group than in the CE group (Fisher’s *z*-test: *z* = 2.99, *p* = 0.003). Sensitivity analysis using Cook’s distance identified one influential observation in the DRE group (age 4 years; Cook’s D = 0.253); its removal strengthened the correlation (ρ = 0.671, *p* < 0.001, *n* = 26), confirming that the finding was not driven by a single data point.

## 4. Discussion

In most children with epilepsy, seizures can be effectively controlled and cognitive function preserved. In others, however, seizures persist despite adequate treatment and are accompanied by memory difficulties, cognitive decline, and other neurological comorbidities [1,2,3,4]. These divergent outcomes raise an important question: do recurrent seizures contribute to progressive neuronal injury and neurodegeneration over time? Identifying neuronal damage through reliable biomarkers could not only improve disease monitoring and guide treatment decisions, but also shed new light on the underlying pathophysiology. In the present study, we compared serum levels of NO-related metabolites, GFAP, and UBI—candidate biomarkers associated with neural injury and neuroinflammation—between children with controlled and drug-resistant epilepsy. We found no statistically significant differences in these serum biomarkers between the CE and DRE groups. The higher seizure frequency, greater diversity of seizure types, more frequent seizure capture on EEG, and elevated comorbidity rates in the DRE group were expected findings, consistent with the existing literature [15].

The role of NO in epileptogenesis and seizure generation has been demonstrated in various experimental studies, with both proconvulsant and anticonvulsant effects reported depending on the model and conditions [6,7,8,9]. Clinical data also point to alterations in NO metabolism in childhood epilepsy. Arhan et al. reported significantly elevated serum NO levels in newly diagnosed epileptic children compared to healthy controls, suggesting that NO dysregulation may emerge early in the epileptogenic process [16]. In our study, however, no significant difference in serum NO-related metabolite levels was found between the CE and DRE groups. As our analysis was limited to a comparison between these two patient groups, we were unable to determine whether NO-related metabolite levels were elevated relative to a healthy pediatric population.

Of note, the positive correlation between age and serum NO-related metabolites was observed exclusively in the DRE group, with no such association in the CE group. Formal comparison of the two correlation coefficients confirmed that this association was statistically significantly stronger in the DRE group than in the CE group (Fisher’s *z*-test: *z* = 2.99, *p* = 0.003). This group-specific association is hypothesis-generating and may reflect differences in age- or disease-related NO metabolism; however, given the cross-sectional design and potential confounding by epilepsy duration, seizure burden, comorbidities, and ASM exposure, its underlying mechanism cannot be established, and the finding does not demonstrate progressive NO upregulation or cumulative neuroinflammatory burden.

The biological properties of NO add another layer of complexity to this interpretation. Because it is rapidly converted to nitrite and nitrate, peripheral serum concentrations of NO-related metabolites may not accurately reflect local NO production within the central nervous system. Circulating levels should therefore be interpreted as an indirect estimate of NO-related biological activity rather than a direct measure of free NO [17].

GFAP is widely recognized as one of the most important biomarkers of astroglial injury and reactive astrogliosis [11,18]. Recurrent and refractory seizure activity is known to trigger reactive astrogliosis, leading to upregulation of GFAP expression in astrocytes. In the context of chronic epileptiform activity, disruption of blood–brain barrier integrity and structural changes in perivascular astroglial end-feet may facilitate intermittent or transient leakage of GFAP into the systemic circulation, which would support the expectation of chronically elevated circulating levels in drug-resistant epilepsy [19]. In our study, however, serum GFAP levels did not differ significantly between the CE and DRE groups, a finding that does not support persistently elevated circulating GFAP levels in childhood DRE. Both Aksoy et al. and Elhady et al. reported significantly elevated serum GFAP levels in children with epilepsy, particularly in those with active and frequent seizures [10]. Similarly, Simani et al. demonstrated that serum GFAP was significantly higher in patients with epileptic seizures compared to those with psychogenic non-epileptic seizures, particularly when samples were collected within the first six hours after a seizure [20]. Dobson et al. likewise found elevated plasma GFAP in patients with epilepsy compared to those with non-epileptic events [21]. These findings suggest that circulating GFAP levels may be influenced by the temporal proximity of blood sampling to seizure activity.

In our study, blood samples were not collected at a standardized interval after the last seizure. Despite the higher seizure frequency in the DRE group, GFAP levels were comparable between groups. Our results are in line with the findings of Akel et al., who demonstrated in a large regional epilepsy cohort that elevated plasma GFAP is not a feature of the chronic interictal state but rather a transient, seizure-related phenomenon [22]. Together, these findings suggest that circulating GFAP may be more closely associated with acute seizure-related changes than with chronic disease severity. The absence of a healthy control group in our study may also have limited our ability to detect differences in GFAP levels that were not apparent in the comparison between the CE and DRE groups.

The ubiquitin system is one of the principal mechanisms regulating intracellular protein degradation and plays a critical role in maintaining neuronal homeostasis. Ubiquitin-mediated proteolysis is known to be involved in cellular responses to neuronal injury [5]. In our study, serum ubiquitin (UBI) concentrations did not differ significantly between the CE and DRE groups. Clinical evidence regarding circulating ubiquitin itself in epilepsy remains limited, which restricts direct comparison of our findings with previous studies.

Several previous epilepsy biomarker studies, including those by Aksoy et al. and Asadollahi and Simani [23,24], investigated ubiquitin C-terminal hydrolase L1 (UCH-L1) rather than ubiquitin itself. UCH-L1 is a neuron-enriched deubiquitinating enzyme involved in regulation of the ubiquitin–proteasome system, but it is biologically distinct from ubiquitin. Therefore, findings regarding circulating UCH-L1 and its postictal kinetics cannot be directly extrapolated to serum ubiquitin.

Although the interval between the most recent seizure and blood sampling was not standardized or systematically recorded in our study, the implications of sampling time for circulating ubiquitin cannot be determined from UCH-L1 studies. Therefore, our finding should be interpreted conservatively as showing that no statistically significant difference in serum ubiquitin concentrations was detected between the CE and DRE groups in this cohort. Further studies specifically evaluating circulating ubiquitin, with standardized sampling relative to seizure timing and appropriate healthy controls, are needed to clarify its potential role as a peripheral biomarker in childhood epilepsy.

## 5. Limitations

The most significant limitation is the absence of the originally planned healthy-control group. Thirty healthy controls had been recruited; however, their serum samples, which were stored separately from the CE and DRE samples, were rendered unsuitable for analysis following a deep-freezer malfunction and subsequent thawing. The CE and DRE samples were stored in a different deep-freezer and were not affected by this event. Consequently, the originally planned comparison with healthy controls could not be performed, and the final analysis was restricted to comparisons between the CE and DRE groups. This limits our ability to determine whether biomarker levels in either epilepsy group differ from those in a healthy pediatric population.

A further limitation concerns the timing of blood sampling. The interval between the most recent clinical seizure and blood collection was not systematically recorded, precluding reliable classification of samples as interictal, peri-ictal, or postictal. Determining the exact timing of the most recent seizure is also inherently difficult in clinical practice, as it relies on parental report, is subject to recall bias, and may be further complicated by unwitnessed seizures. Subclinical seizure activity, particularly in patients with drug-resistant epilepsy, adds another layer of difficulty to the interpretation of serum biomarker levels.

Another limitation concerns ASM exposure. Restricting the CE group to patients controlled with a single ASM reduced treatment-related heterogeneity but may have introduced selection bias by excluding seizure-free children receiving two or more ASMs. In addition, the DRE group had a substantially greater and more heterogeneous medication burden than the CE group. Because ASMs may influence oxidative, nitrosative, inflammatory, and cellular stress pathways, differences in medication exposure may have confounded circulating biomarker concentrations. Although information on previous ASM use was available from clinical records, cumulative exposure to previously used ASMs could not be consistently quantified in terms of treatment duration and dose. Given the relatively small DRE sample and the heterogeneity of ASM combinations, robust multivariable adjustment for individual ASM classes and other clinical covariates was not feasible; therefore, residual confounding by treatment burden and other clinical factors cannot be excluded. Pubertal status was not systematically recorded and therefore could not be evaluated as a potential confounder of biomarker concentrations.

Finally, no formal a priori sample size calculation was performed, as this was an exploratory cross-sectional study; the sample comprised all consecutive eligible patients during the recruitment period. The relatively small DRE group (*n* = 27) limits statistical power, and the wide confidence intervals for the effect size estimates reflect this limited precision. The cross-sectional design of the study did not allow us to track changes in biomarker levels over time or capture dynamic fluctuations associated with seizure occurrence. Longitudinal measurements would be needed to characterize the temporal relationship between seizure activity and peripheral biomarker concentrations. In addition, this was a single-center study conducted at a tertiary referral center and included only children with epilepsy of unknown etiology. Therefore, the findings should be interpreted cautiously and may not be generalizable to children with other etiological forms of epilepsy or to broader pediatric epilepsy populations.

## 6. Conclusions

Serum levels of three candidate biomarkers associated with neural injury and neuroinflammation (GFAP, UBI, and NO-related metabolites) did not differ significantly between children with controlled and drug-resistant epilepsy in this cohort. A positive association between age and NO-related metabolite levels was observed in the DRE group; however, given the cross-sectional design and potential confounding by epilepsy duration, seizure burden, comorbidities, and ASM exposure, this finding should be interpreted cautiously and does not establish progressive NO upregulation or cumulative neuroinflammatory burden. The findings should also be interpreted in light of the absence of a healthy control group, the modest sample size, differences in ASM exposure between groups, and the lack of standardized sampling relative to the most recent seizure. Future longitudinal studies including healthy controls, standardized seizure-to-sampling intervals, and adequately powered analyses accounting for relevant clinical and treatment-related confounders are needed to determine the potential utility of these candidate peripheral biomarkers in childhood epilepsy.

## Figures and Tables

**Table 1:** Demographic and Clinical Characteristics of the Study Groups.

Parameter	DRE (*n* = 27)	CE (*n* = 58)	*p*-Value
Sex, n (%)			0.093
Female	15 (55.6)	21 (36.2)	
Male	12 (44.4)	37 (63.8)	
Age (years), median (IQR)	10.0 (7.5–13.0)	12.0 (9.0–14.0)	0.110
Birth weight, n (%)			0.850
<2500 g	2 (7.4)	5 (8.6)	
2500–4000 g	25 (92.6)	53 (91.4)	
Maturity status, n (%)			0.766
Preterm	1 (3.7)	3 (5.2)	
Term	26 (96.3)	55 (94.8)	
NICU admission history, n (%)	4 (14.8)	3 (5.2)	0.201
Family history of febrile convulsion, n (%)	1 (3.7)	8 (13.8)	0.261
Family history of epilepsy, n (%)	3 (11.1)	17 (29.3)	0.066
Consanguinity, n (%)			0.803
None	24 (88.9)	48 (82.8)	
First-degree	2 (7.4)	5 (8.6)	
Second-degree	1 (3.7)	5 (8.6)	
Comorbidity, n (%)	19 (70.4)	21 (36.2)	0.003
Type of comorbidity, n (%)			0.004
None	8 (29.6)	37 (63.8)	
Learning disability	4 (14.8)	10 (17.2)	
Autism	3 (11.1)	1 (1.7)	
ADHD	1 (3.7)	0 (0.0)	
Multiple comorbidities	11 (40.7)	10 (17.2)	

There was no significant difference between the groups regarding duration of epilepsy or EEG findings (*p* > 0.05).

**Table 2:** Clinical Characteristics of the Study Groups.

Parameter	DRE (*n* = 27)	CE (*n* = 58)	*p*-Value
Duration of epilepsy (years), median (IQR)	5.0 (3.0–8.0)	4.0 (2.0–7.0)	0.422
Historical seizure frequency, n (%)			<0.001
Rare	0 (0.0)	50 (86.2)	
Persistent	19 (70.4)	8 (13.8)	
Daily	8 (29.6)	0 (0.0)	
Seizure classification, n (%)			0.040
Focal	4 (14.8)	7 (12.1)	
Generalized	20 (74.1)	51 (87.9)	
Multiple seizure types	3 (11.1)	0 (0.0)	
Seizure semiology, n (%)			0.030
Motor	17 (63.0)	46 (79.3)	
Non-motor	7 (25.9)	12 (20.7)	
Motor and non-motor	3 (11.1)	0 (0.0)	
EEG findings, n (%)			0.272
Focal	13 (48.1)	25 (43.1)	
Generalized	14 (51.9)	27 (46.6)	
Normal	0 (0.0)	6 (10.3)	
Seizure detection on initial video-EEG, n (%)			<0.001
None	6 (22.2)	51 (87.9)	
Electroclinical seizure	10 (37.0)	7 (12.1)	
Electrical seizure	7 (25.9)	0 (0.0)	
Both	4 (14.8)	0 (0.0)	

Note: Seizure-frequency data represent historical seizure frequency before seizure control was achieved in the CE group and do not indicate ongoing seizure activity at the time of study enrollment or blood sampling. EEG findings were derived from the initial 1-h video-EEG recordings performed during clinical evaluation and do not represent EEG recordings obtained during the study period.

**Table 3:** Comparison of Serum Biomarker Levels Between the Study Groups.

Biomarker Levels	DRE (*n* = 27)	CE (*n* = 58)	*p* Value	r (95% CI)
NO-related metabolites (µmol/L)	53.08 (41.01–61.56)	48.38 (38.58–59.26)	0.713	−0.050 (−0.306 to 0.211)
GFAP (ng/mL)	7.35 (5.65–8.59)	7.58 (5.93–9.72)	0.759	0.042 (−0.219 to 0.298)
UBI (pg/mL)	248.2 (210.4–346.5)	297.4 (230.5–366.6)	0.199	0.174 (−0.089 to 0.415)

## Data Availability

The raw data supporting the conclusions of this article will be made available by the authors on request.

## Supplementary Materials

The following supporting information can be downloaded at: https://www.mdpi.com/article/10.3390/children13091190/s1, Table S1: Current antiseizure medication exposure according to epilepsy group.
