# Supplementary material for: Candidate Serum Biomarkers of Neural Injury and Neuroinflammation in Childhood Epilepsy: Comparison Between Controlled and Drug-Resistant Phenotypes
Source: Children (Basel). 2026 Sep 3;13(9):1190. doi: 10.3390/children13091190 (PMC13604986; doi:10.3390/children13091190)
Supplement: Supplementary file 1 [file children-13-01190-s001.zip › children-4517164-supplementary.pdf]

Table S1. Current antiseizure medication exposure according to epilepsy group

Antiseizure medication	CE (n = 58), n (%)	DRE (n = 27), n (%)
Valproate (VPA)	23 (39.7)	21 (77.8)
Levetiracetam (LEV)	21 (36.2)	17 (63.0)
Clobazam	1 (1.7)	12 (44.4)
Carbamazepine	10 (17.2)	1 (3.7)
Topiramate	0 (0.0)	10 (37.0)
Lamotrigine (LTG)	2 (3.4)	8 (29.6)
Zonisamide (ZNS)	0 (0.0)	3 (11.1)
Sulthiame (SLTH)	0 (0.0)	3 (11.1)
Phenobarbital (FB)	1 (1.7)	2 (7.4)
Vigabatrin (VGB)	0 (0.0)	2 (7.4)
Lacosamide (LAC)	0 (0.0)	2 (7.4)
Clonazepam	0 (0.0)	1 (3.7)
Ethosuximide (ETX)	0 (0.0)	1 (3.7)

Note: Data are presented as n (%). Percentages may exceed 100% because participants in the DRE group received multiple ASMs concurrently. CE, controlled epilepsy; DRE, drug-resistant epilepsy; ASM, antiseizure medication; ACTH, adrenocorticotropic hormone.

Treatment burden: all CE participants received monotherapy. All DRE participants received at least three ASMs; 25/27 (92.6%) received three ASMs and 2/27 (7.4%) received four ASMs (mean current ASM burden, 3.07 per patient).
